# Insights on Natural Products Against Amyotrophic Lateral Sclerosis (ALS)

**DOI:** 10.2174/1570159X22666231016153606

**Published:** 2024-10-17

**Authors:** Kadja Luana Chagas Monteiro, Marcone Gomes dos Santos Alcântara, Thiago Mendonça de Aquino, Edeildo Ferreira da Silva-Júnior

**Affiliations:** 1 Research Group on Therapeutic Strategies - GPET, Laboratory of Synthesis and Research in Medicinal Chemistry - LSPMED, Institute of Chemistry and Biotechnology, Federal University of Alagoas, 57072-970, Maceió, Alagoas, Brazil;; 2 Institute of Chemistry and Biotechnology, Federal University of Alagoas, 57072-970, Maceió, Brazil

**Keywords:** Central nervous system, reactive oxygen species, ALS, mtDNA, heat shock proteins, medicinal herbs

## Abstract

Amyotrophic lateral sclerosis (ALS) is a progressive neurodegenerative disease that causes the death of motor neurons and consequent muscle paralysis. Despite many efforts to address it, current therapy targeting ALS remains limited, increasing the interest in complementary therapies. Over the years, several herbal preparations and medicinal plants have been studied to prevent and treat this disease, which has received remarkable attention due to their blood-brain barrier penetration properties and low toxicity. Thus, this review presents the therapeutic potential of a variety of medicinal herbs and their relationship with ALS and their physiopathological pathways.

## 1 INTRODUCTION

Amyotrophic lateral sclerosis (ALS) is a progressive neurodegenerative disease that causes neuron death in the cerebral motor cortex, brainstem, and spinal cord, restricting voluntary movements. Besides, ALS patients present difficulty in speaking, swallowing, and breathing caused by muscle paralysis [1]. Under normal conditions, the nervous impulse transmits messages from brain neurons (upper motor neurons) to spinal cord motor neurons (lower motor neurons) and posteriorly to each muscle [2]. In ALS, both upper and lower neurons degenerate or die and stop sending messages to the muscles, which leads to a gradual weakening of the musculature, which twitches until it atrophies. In cases involving the diaphragm and chest wall, the patient loses the ability to breathe and may experience respiratory failure, often fatal [3, 4]. There is no treatment available to cure and effectively reverse the progression of the disease, which may be related to its multi-faceted physiopathological mechanism [5]. Current treatment options are Riluzole and Edavarone, based on symptoms and respiratory support with only modest benefits. Significant genetic components and variants that cause or predispose ALS development remain unknown, which causes further delays in the development of new treatments [6]. With this obscure perspective, several alternatives have been taken into account in searching for new agents that could act on this disease. Interestingly, natural products have gained extensive importance as alternative therapeutic, as in most cases, they have low toxicity and high efficiency [7, 8]. Natural products originating from plants, animals, marine organisms, and microorganisms have long been an invaluable potential resource for discovering lead compounds and novel drugs [9]. These present many oxygen atoms, *sp*^3^-hybrid bridged or spiro-ring carbon atoms, and chiral centers, which differ from the structural backbones of synthetic compounds [10, 11]. These characteristics make modulation of multiple cellular signaling pathways easier and bind to multiple targets with selectivity [12]. As for neurodegeneration, when compared with synthetic and small molecules, a large number of natural products inhibit neuroinflammation and degeneration but also participate in the promotion of injured-neuron repair in some cases [13, 14]. The investigation of natural products can finally provide relevant structural information to be added to studies of rational planning of new drugs. Furthermore, new therapeutic approaches, such as adjuvant treatments in conjunction with synthetic molecules, may provide better results than those observed with isolated molecules [15, 16]. Currently, a variety of mechanisms are associated with the progression of neuronal degeneration in ALS. However, oxidative stress, excitatory amino acid toxicity, neuroinflammation, and calcium cytotoxicity are more frequently associated with natural products active against ALS.

## 2 OXIDATIVE STRESS AND ALS

The production of reactive oxygen species (ROS) and reactive nitrogen species (RNS) is mainly secondary to enzymatic reactions involved in the respiratory chain, Cytochrome P-450 (CYP450) activity, prostaglandin synthesis, and phagocytosis [17]. Not surprisingly, the production of ROS is a key factor in the etiology of several neurodegenerative diseases [18]. However, a moderate increase of ROS secondary to mitochondrial activity produces preconditioning, which leads to a neuroprotective function against harmful agents [19, 20]. The role of cellular protection in response to ROS accumulation has been reported, and the signal of preconditioning that leads to this protection was related to Hormesis, which means a dose-response relationship of ROS, at low levels, generates a stimulus of neurodegenerative diseases, while its accumulation (high concentration) generates an inhibition of the effect through preconditioning [21-23].

These reactive species play an important role in redox signaling, in particular, ROS (O_2_^-^ and H_2_O_2_), where through structural changes in the cysteine residues of proteins andiron-sulfur cluster proteins, it promotes the functioning of physiological signaling pathways that regulate processes of immunity, thermogenesis, aging, cognition, steroidogenesis, development and proliferation [24, 25].

**Fig. (1):**
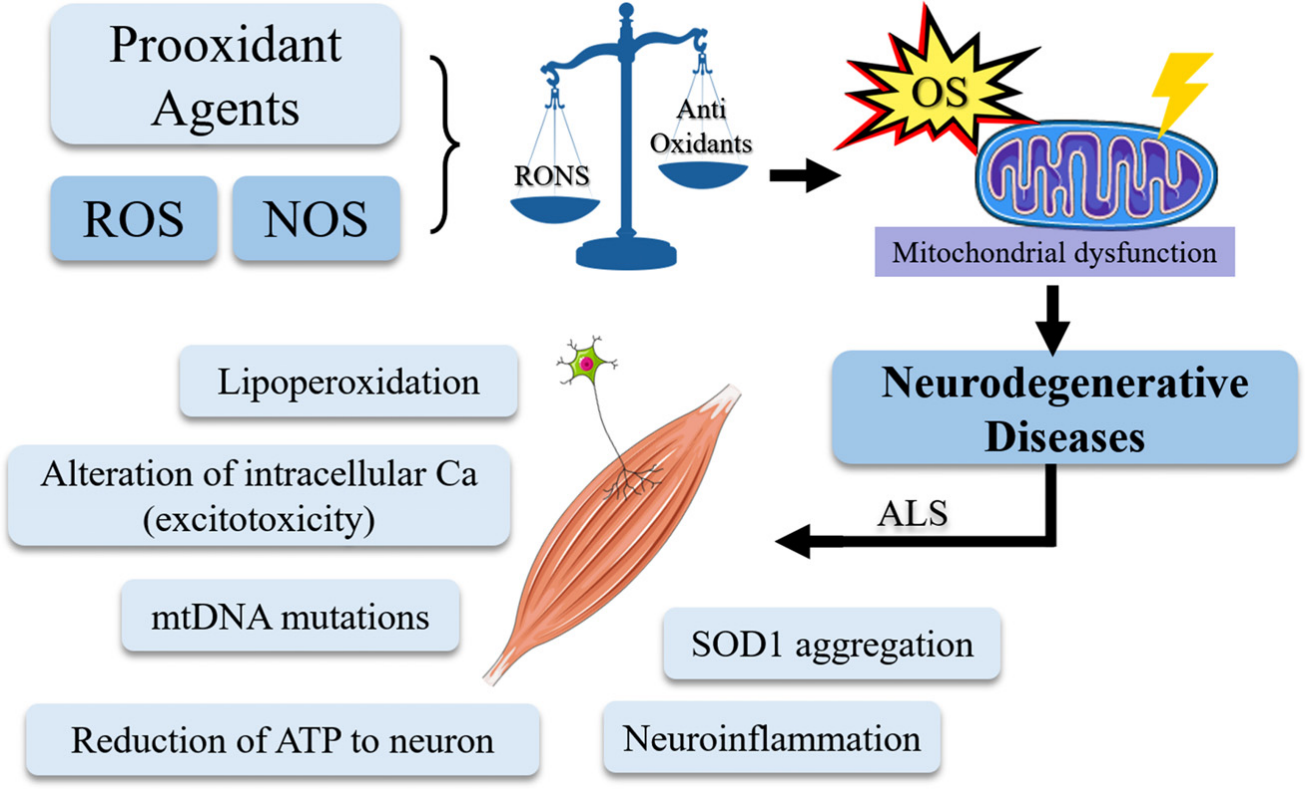
Oxidative stress is caused by an imbalance between antioxidant defenses and RONS. OS and mitochondrial dysfunction have been identified as mechanisms involved in the pathogenesis of ALS and are related to neuroinflammation, aggregation of TDP-43, Lipoperoxidation, mutations in the mtDNA, aggregation of SOD1, and reduction of ATP to neurons.

Although, the problem lies in impaired redox homeostasis, which causes damage to the cell membrane, compromising the viability and integrity of the central nervous system (CNS). Mitochondrial damage compromises the ATP energy supply to neurons and calcium homeostasis. It leads to increased ROS levels, which in turn promotes mitochondrial DNA (mtDNA) mutation and lipid peroxidation of neuronal membranes [26, 27]. The accumulation of mtDNA causes an increase in oxidative damage, which leads to a decrease in the energetic rate and the production of more ROS (Fig. **1**). This entire process of mitochondrial dysfunction is responsible for a series of neuronal damage, genetic mutations, and metabolic stress, which invariably can lead to apoptosis [28]. With regard specifically to ALS pathogenesis, it was discussed the role of antioxidant enzymes (*i.e*., metalloenzymes) as an inactivating agent of free oxygen radicals, converting them into a less harmful substance. The antioxidant enzyme SOD1 is mutated in the familial form of ALS and up to 7% of sporadic ALS [29].

Biomarkers of oxidative stress (OS) and high levels of ROS were observed in the CNS in ALS patients [30], in addition to certain types of ROS (H_2_O_2_ and O_2_^•-^) in affected cells [31]. Mutant forms of SOD1, an antioxidant enzyme that catalyzes the conversion of O_2_^•-^ to H_2_O_2_ and O_2_, which is critical in OS regulation [32, 33], have been associated with loss of oxidative control and excessive production of ROS [34-36]. Thus, OS contributes to SOD1 aggregation, increasing mitochondrial dysfunction [37, 38]. Other significant oxidative changes, such as low levels of reduced glutathione (GSH) in erythrocytes and the motor cortex [39, 40] and a systemic pro-inflammatory state [41] in ALS patients, have been observed. Other studies have shown that oxidative damage to nerve cells, a hallmark of ALS, was related to neurodegeneration [42]. High levels of carbonylated proteins and advanced oxidation products were measured in the motor cortex and plasma of ALS patients [43, 44]. In addition, the relationship between vitagene, oxidative damage, and biological resilience mechanisms was also noted as important in neurodegenerative diseases [45-47]. The importance of vitagenes lies in their participation in the nuclear factor erythroid 2-related factor (Nrf2) antioxidant pathway. They play a crucial role in tissue maintenance and repair by releasing antioxidant proteins known as heat shock proteins (Hsp), including Hsp32, Hsp70, thioredoxin, and the sirtuin protein system. These proteins effectively combat different types of stress, such as oxidative and proteotoxic stress, as well as environmental and neurotoxic effects caused by reactive nitrogen species (RNS) or nitric oxide (NO) [21, 22, 45, 48].

**Fig. (2):**
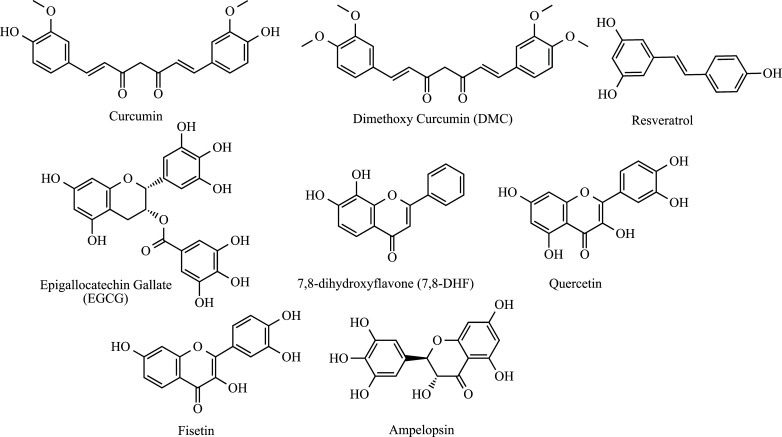
Phytochemicals antioxidant agents in ALS: Curcumin, DMC, Resveratrol, EGCG, 7,8-DHF, Quercetin, Fisetin, and Ampelopsin.

Phytotherapeutic oxidizing agents can regulate oxidative stress, improve the antioxidant activity of various enzymatic and non-enzymatic systems, and maintain the expression and regulation of genes involved in ALS [49, 50]. Curcumin (Fig. **2**) is a natural and liposoluble polyphenolic dye obtained from the crude rhizome of *Curcuma longa*. An expressive amount of studies on the anti-inflammatory and neuroprotective effects have been explored [51, 52]. An important study by Jiang *et al.* demonstrated that curcumin activates the nuclear factor Nrf2, which is a master transcriptional regulator of phase II antioxidant genes. The authors reported that curcumin led to a significant induction of phase II enzymes, which play an important role in protecting cells against stress through the removal of free radicals and detoxication. Thus, the activation of Nrf2 target genes promoted a reduction in the levels of ROS and attenuated oxidative damage and mitochondrial dysfunction [53]. It was later displayed that curcumin can even eliminate the excitability induced by the TAR DNA-binding protein 43 (TDP-43) [54]. Mutation of this protein is detected in patients with familial and sporadic ALS (ALSf and ALSs) [55, 56]. Dimethoxy Curcumin (DMC) (Fig. **2**) also demonstrated significant effects on the TDP-43 protein, improving mitochondrial dysfunction in mutated TDP-43 stably transfected cell lines [57]. As already mentioned, ALS has a direct relationship with the pathological deposition of superoxide dismutase (SOD1). Thus, a strategy to combat ALS consists of inhibiting the formation of SOD1. With this objective, the study by Bhatia *et al.* demonstrated that curcumin inhibits DTT-induced SOD1 fibrillation and favors the formation of smaller and disordered SOD1 aggregates, in addition to a reduction in SOD1-mediated toxicity [58]. Recent work showed the wild-type TDP-43 increased the firing frequency of action potentials, and the mutant Q331K TDP-43 enhanced the firing frequency and decreased the threshold of action potentials to a higher level. Also, the mutant and wild-type TDP-43 induced a more rapid speed of recovery from fast and slow inactivation of the Nav channel, reducing the voltage dependency of slow inactivation [54]. DMC and curcumin significantly decrease the abnormities of action potentials and Nav channels [54]. Curcumin has shown positive neuroprotection results in preclinical *in vitro* and *in vivo* studies [59]. However, its low bioavailability due to limited absorption and rapid metabolism results in low serum concentrations and has been a critical point in its clinical development [60, 61]. To overcome this limitation, Tripodo *et al.* propose a drug delivery system formed by a carrier-in-carrier device for systemic delivery, targeting hydrophobic drugs mediated by micelle-loaded mesenchymal stromal cells to be administered by intravenous injection [62]. The curcumin-loaded micelles, sterilized by filtration, reached the maximum loading in mesenchymal stromal cells in a few minutes, and compared to free curcumin, an evident reduction of cytotoxicity on mesenchymal stromal cells was detected, demonstrating that this is an innovative drug delivery system. A study where rats were exposed to 500 ppm curcumin through diet for 4 weeks showed that supplementation with curcumin in the diet reduced the action of oxidative stress and might counteract the deleterious effects of traumatic brain injury [63]. Other studies have also been successful in improving the bioavailability and pharmacokinetic characteristics of curcumin [64, 65]. In a double-blind, randomized, placebo-controlled trial conducted for 12 months, patients with a definite or probable ALS diagnosis were randomly assigned to receive either nano curcumin (80 mg per day) or placebo, in add-on therapy to Riluzole. The treatment showed a reduction in death or dependence on mechanical ventilation (18% difference compared to the placebo group), which suggests that nano curcumin might improve the probability of survival as an add-on treatment, especially in patients with existing bulbar symptoms [66]. In another double-blind therapeutic trial, curcumin treatment shows encouraging results, demonstrating a slight slowdown in disease progression, improving aerobic metabolism and oxidative damage [50]. The effect of curcumin on microglial cells has also been studied and deserves notable attention, especially in tissue repair [67].

Continuing our discussion of polyphenols, these can be classified into different classes according to the number of phenolic rings in their structure and the substituents linked to the rings. There are four groups: phenolic acids, phenolic diterpenes, flavonoids, and volatile oils [68]. Resveratrol (3,5,4′-trihydroxystilbene) (Fig. **2**) is a stilbenoid analog found in grapes, berries, peanuts, and others. Despite its low oral bioavailability, resveratrol has a variety of biological actions, including antioxidant and neuroprotective. It has been shown that resveratrol has positive effects by up-regulating sirtuin 1 (SIRT1) expression in the mutant SOD1^G93A^-bearing motor neuron-like culture model of ALS, improving the cell viability and ATP levels and preventing cell apoptosis [69]. Similarly, the antioxidant activity of resveratrol has previously been reported to be protective against ALS [70]. Mancuso *et al.* demonstrated that resveratrol exerts potent therapeutic actions in the SOD1^G93A^ model of ALS [71]. The treatment significantly delayed disease onset and extended animal survival. Resveratrol exerted neuroprotective effects mainly through increasing the expression of SIRT1, suppressing oxidative stress, and downregulating p53 and its related apoptotic pathway [72]. In another complementary study, the effect of the combination of resveratrol and a sigma-1R agonist selective agent was evaluated. The combined treatment significantly ameliorated SOD1^G93A^ mice, but it did not show a synergistic effect compared to treatment with isolated substances [73]. Similarly, Srinivasan *et al.* demonstrated that resveratrol treatment attenuated motor neuron loss, relieving muscle atrophy, and improved the mitochondrial function of muscle fibers in the SOD1^G93A^ mouse model of ALS. The interaction between resveratrol and the mutant form SOD1^G93A^ has also been evaluated by quantum chemical and molecular mechanics approaches [74]. In another study, researchers revealed that bone marrow mesenchymal stem cells extracted from ALS patients exhibited a decrease in AMPK/SIRT1 signaling, which was subsequently restored through resveratrol treatment [75]. Recently, it was observed that the resveratrol treatment significantly reduced thimerosal-induced neurotoxicity in SOD1^G93A^ cells [76]. Hydroxytyrosol (HT) has received notable attention for its antioxidant properties [77-79]. This important component of the Mediterranean diet has been associated with increased survival and motor performance in transgenic mice that overexpress the human variant SOD1^G93A^ [80]. This result was associated with the expression of myogenic factors and autophagy markers, in addition to a decrease in endoplasmic reticulum stress [80, 81]. Recently, a review addressed the potential of Genus *Boswellia* in the study of neurodegenerative disorders [82]. Although its direct relationship with ALS is new, Boswellic acid has been associated with anti-oxidant and anti-inflammatory properties that may be useful in ALS-related studies [83, 84]. Recently, Boswellic acid has been linked to neuroprotective effects against an experimental model of ALS induced by methylmercury (MeHg). It achieves this by modulating the Nrf2/HO-1 signaling pathway and facilitating the release of antioxidant and anti-inflammatory proteins. Consequently, it helps alleviate neuroinflammation and promotes remyelination [85].

Epigallocatechin gallate (EGCG) (Fig. **2**) is another water-soluble polyphenol isolated from green tea [86]. Several pharmacological activities have been attributed to this compound, such as anticancer, antioxidant, and neuroprotective effects. These attributes are attributed to its capacity to traverse the blood-brain barrier and regulate the mitochondrial response to oxidative stress (OS) [87]. One of the first insights was reached by the group of Hockenbery *et al.* It was observed that the upregulation of the Bcl-2 gene, which inhibits most types of apoptotic cells, and the downregulation of the *Bax* gene, which can promote apoptosis, were detected in cells treated with EGCG [88]. It was later observed that EGCG promotes protection against lipid peroxidation, highlighting its *in vitro* antioxidant effects [89]. The neuroprotective potential of EGCG against OS-induced cell death was also reported through the restored reduced protein kinase C (PKC) and extracellular signal-regulated kinases (ERK1/2) activities [90, 91]. In another oxidative stress-induced apoptosis model, 6-Hydroxydopamine (6-OHDA) was used as an inducer in catecholaminergic PC-12 cells, and significant neuroprotective effects against apoptosis were reported, even better than the green tea polyphenol mixture [92]. This result was later reinforced in another study with the same cells [93]. Equally important, it has been shown that the EGCG can prevent OS-induced death of mutant SOD1 motor neuron cells by alteration of cell survival and death signals [94, 95]. In their first study, Koh *et al.* observed that EGCG demonstrates neuroprotective effects by upregulating PI3K/Akt and GSK-3 pathways in G93A mutant cells [96]. Later, the group evaluated the effect of ECGC in ALS model mice with the mutated gene SOD1^G93A^. Treatment was observed to significantly prolong symptom onset and lifespan, in addition to preserving signs of survival and reducing signs of death [97]. Similarly, a study performed by Xu *et al.* demonstrated the neuroprotective effects of EGCG in a transgenic mouse model of ALS SOD1^G93A^. Oral administration of EGCG from a pre-symptomatic stage has been shown to delay disease onset and prolong life span, in addition to showing an increased number of motor neurons, decreased microglial activation, reduced immunohistochemical reaction of NF-κB, and cleaved caspase-3, as well as, reduced protein levels of iNOS and NF-κB in the spinal cord [98]. More recently, a computational study was performed to examine the inhibitory action of EGCG against native and mutant SOD1, demonstrating that EGCG reduced the formation of toxic aggregates after mutation [99].

Many studies have been carried out to highlight the potential of flavonoids and polyphenols as antioxidant agents [100-104], including those for neurodegenerative diseases [105, 106]. In this class, 7,8-dihydroxyflavone (7,8-DHF) (Fig. **2**) showed neuroprotective and neuromuscular transmission regulatory properties. This flavonoid selectively activates enhanced neuromuscular transmission *via* TrkB activation in the diaphragm muscle [107]. Later, the neuroprotective effects of 7,8-DHF in a transgenic ALS mouse model SOD1^G93A^ were evaluated, demonstrating that chronic administration of 7,8-DHF significantly improved motor deficits and preserved spinal motor neurons count and dendritic spines [108]. Similarly, Quercetin (Fig. **2**) has been shown to reduce OS-induced mitochondrial damage. As demonstrated in the work of Sharma *et al.*, quercetin was able to reduce aluminum-induced oxidative stress in the rat hippocampus. In addition, they demonstrated that quercetin also prevents aluminum-induced translocation of cytochrome *c*, upregulates Bcl-2, down-regulates *Bax*, *p53*, and caspase-3 activation, and reduces DNA fragmentation [109]. It was later shown that quercetin and its derivative quercetin-3-*β*-*D*-glucoside were found as inhibitors of misfolding and aggregation in SOD1-associated ALS [110]. Fisetin (Fig. **2**) was also recently cited as a new protector against ROS damage. Research has indicated that it offers neuroprotection with an improved survival rate, attenuated motor impairment, reduced ROS damage, and regulated redox homeostasis [111]. Furthermore, fisetin increased the expression of phosphorylated ERK, increased the expression of antioxidant factors, and reduced the levels of mutant and wild-type SOD1 [111].

Ampelopsin (Fig. **2**) is a flavonoid isolated from *Ampelopsis grossedentata*, which is known to have antioxidant properties that can be studied in neurodegenerative processes [112, 113]. A pioneering study by Kou *et al.* demonstrated the potent antioxidant activity of Ampelopsin in PC-12 cells that underwent H_2_O_2_-induced apoptosis. The 1-hour treatment reduced vitality loss, inhibited ROS formation and prevented H_2_O_2_-induced *p38* activation. Additionally, the treatment was effective in upregulating heme oxygenase-1 (HO-1) due to the activation of ERK and Akt signaling pathways [114]. Other natural oxidants related to the treatment of ALS have been reported. *Ginkgo biloba* L. or maidenhair is a well-known plant belonging to the *Ginkgoaceae* family, which has in its extract some flavonoid glycosides such as myricetin, kaempferol, and the aforementioned quercetin [115]. A standardized extract of *Ginkgo biloba* showed protective effects against mitochondrial damage and OS in a transgenic mouse model of ALS. The extract significantly improved motor performance and survival and protected against a loss of spinal-cord anterior motor horn neurons in male mutant transgenic ALS mice but not in littermate females [116]. Other studies have also reported the effects of Ginseng [117] and Genistein [118] in a transgenic mouse model of ALS.

**Fig. (3):**
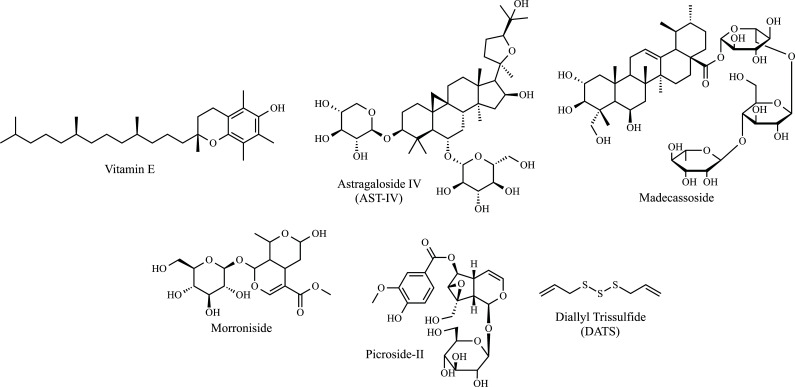
Phytochemicals antioxidant agents in ALS: Vitamin E, AST-IV, Madecassoside, Morroniside, Picroside-II, and DATS.

Another OS-modulating substance is Vitamin E (*α*-tocopherol) (Fig. **3**) [119]. A preliminary study showed that dietary supplementation with *α*-tocopherol delays the onset of clinical disease and slows progression in transgenic mice that express mutated copies of the gene encoding SOD1 [120]. In a double-blind, placebo-controlled, randomized clinical trial, it was shown that after 12 months of treatment, vitamin E administration did not influence survival and motor function in ALS. The utilization of an association with riluzole for 3 months allowed us to verify changes in the levels of biochemical markers for oxidative stress [121]. Later, regular use of vitamin E supplements was associated with a lower risk of dying from ALS [122-124]. However, the study performed by Graf *et al.* demonstrated no significant difference between placebo and treatment groups (treated with *α*-tocopherol) [125]. Furthermore, there are not enough results to state that megadoses of vitamin E can effectively reduce the progression of ALS [125-127]. Thus, further studies are needed to analyze the relationship between vitamin E and ALS. Carotenes such as *β*-carotene, astaxanthin, and lycopene have also been related to trials in which the intake of these pigments was associated with a reduced risk of ALS [128-131]. However, further studies should be performed for more conclusive results.

Some structurally diverse substances have also been reported as potent regulators of ROS in neurodegenerative processes. Astragaloside IV (AST-IV) (Fig. **3**) is a saponin isolated from *Radix astragali,* often cited for its antioxidant properties [132, 133]. The study with a traditional Chinese formulation containing *Radix astragali* was carried out by Rong *et al.* The authors mentioned that the formulation's actives act by a cytoprotective mechanism against OS and that *Radix astragali* would be one of those responsible for the induction of HO-1 [134]. Later, it was reported that AST-IV could increase the activity and content of SOD in the cytoplasm, which is important in ALS [135]. AST-IV pretreatment was shown to attenuate the H_2_O_2_-induced loss of SH-SY5Y human neuronal cells in addition to decreasing apoptosis and attenuating ROS overproduction [136]. Recently, the protective effect against neuronal damage caused by microglia has been studied. AST-IV protected microglia from lipopolysaccharide-evoked death and down-regulated the release of pro-inflammatory mediators, including interleukin IL-1*β*, IL-6, tumor necrosis factor *α* (TNF-*α*), and nitric oxide, as well as, expression of Toll-like receptors 4 (TLR4), MyD88, and NF-κB [137].

A saponin associated with antioxidant activity against ALS is Madecassoside (Fig. **3**). It is isolated from *Centella Asiatica*. This triterpenoid saponin was evaluated in an assay in mice expressing SOD1^G93A^, in which it was possible to observe a significant reduction in the decline in motor strength of mice during the slowest decline stage to the final stage of the disease [138]. In a model of hippocampal neuron injury induced by chronic aluminum intoxication, Madecassoside was shown to reduce neuronal damage caused by OS and inhibit apoptosis [139]. Recently, it was reported that the expression of pro-neuroinflammatory genes such as inducible nitric oxide synthase, cyclooxygenase-2, signal transducer, an activator of transcription 1, and nuclear factor-κB, was significantly downregulated in a dose-independent manner following treatment with Madecassoside [140]. In another study, Madecassoside was evaluated in an *in vivo* model for its potential in lipopolysaccharide-induced cognitive impairment and neuroinflammation, revealing that treatment for 14 days with 120 mg/kg reduced neurotoxicity, cognitive impairments and the production of inflammatory cytokine agents such as IL-1*β*, TNF-*α,* and IL-6 by a mechanism of activation of Nrf2 signaling [141].

Natural iridoid glycosides such as Morroniside and Picroside-II (Fig. **3**) have been extensively studied as potentially useful antioxidants in treating neurodegenerative diseases, including ALS. They can be isolated from the dry ripe fruit of *Cornus officinalis Sieb. et Zucc.* (*Cornaceae*) and *Pseudolysimachion rotundum var. subintegrum*, respectively [142, 143]. As demonstrated in the studies performed by Wang *et al.*, morronoside demonstrated inhibitory effects on ROS formation and activation of caspase-3 and 9, in addition to up-regulating Bcl-2 in SH-SY5Y cells of a model of H_2_O_2_-induced toxicity [144]. Using the same model, the group also verified that the morronoside reduced the intracellular accumulation of Ca^2+^ and decreased the mitochondrial membrane potential caused by the addition of H_2_O_2_. Additionally, morronoside significantly inhibited SOD1 activity and the percentage of cells in apoptosis in a dose-dependent manner [145]. After that, the group further reported on the potential of morronoside to protect the brain against damage induced by focal cerebral ischemia, probably related to its antioxidant activity in the brain [146]. A model of H_2_O_2_-induced cell death in SK-N-SH human neuroblastoma cells was evaluated, in which morronoside protected cells from oxidative damage by inhibiting ROS production while suppressing Bcl-2-associated X protein (Bax), stimulated the expression of Bcl-2 and blocking apoptosis [147].

On the other hand, Picroside-II was reported to be responsible for the marked increase in neurite outgrowth in PC-12 cells, activity attributed to picroside-II nerve growth factor (NGF) [148-150]. Two other models were evaluated, one *in vitro* (in PC-12 cells treated with glutamate) and another *in vivo* (in male mice treated with AlCl_3_). In the first, picroside II enabled an increase in cell viability and a reduction in glutamate-induced ROS. While in the *in vivo* model, picroside II markedly improved learning and memory dysfunctions. The authors attribute the positive results to the increase in SOD in the mouse brain, relating this substance to the potential against OS [151].

Diallyl trisulfide (DATS) (Fig. **3**) is an organic sulfide derived from the *Liliaceae allium* plant [152]. It is known for its ability to cross the blood-brain barrier (BBB). DATS treatment activated HO-1, demonstrating a protective role, especially against oxidative and inflammatory damage [153]. DATS has been shown to cause activation of Nrf2 and Nrf2 target genes in rat spinal cord explants and protect motor neurons against glutamate-induced excitotoxicity [154]. Neuroprotective effects were also reported in a model of SOD1^G93A^ transgenic mice. Oral administration of DATS-induced acid protein prolonged duration and extended life span for about one week, in addition to HO-1 and reduced glial fibril expression in the lumbar spinal cord of these transgenic mice [155]. In another work, Liu *et al.* demonstrated the action of DATS against motor neuron-like NSC34 cells overexpressing TDP-43, a pathological and biochemical marker for ALS. The treatment led to induced neuronal autophagy and lysosomal clearance of TDP-43 and C-terminal TDP-43 fragments. It was also observed that the Nrf2 was accumulated in the nucleus, and the expression of HO-1 and NAD(P)H: quinone oxidoreductase was increased [156]. Similarly, activation of Nrf2 was also demonstrated in an *in vivo* cerebral ischemia model, with increased SOD1 in the nucleus, SOD2, glutathione S-transferase and peroxidase in the cortex, and increased activity of catalase in the striatum, evidencing the protective effects of DATS [157]. All this evidence suggests the important role of antioxidant agents in neurodegenerative diseases, especially those associated with ALS [158, 159].

## 3 NEUROINFLAMMATION AND ALS

**Fig. (4):**
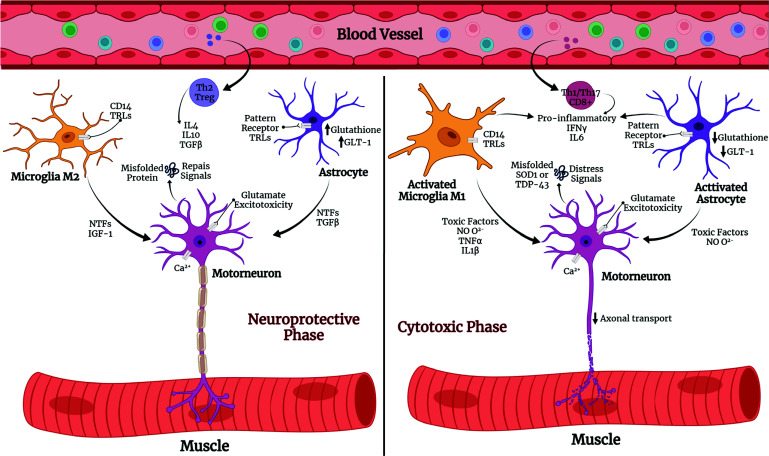
Representation of the neuroprotective and cytotoxic phase of neuroinflammation. During the initial periods of disease progression (neuroprotective phase), the compensatory response is governed by T helper 2 cells (Th2)/regulatory T-lymphocytes (Tregs), Microglia M2, and astrocytes secreting neurotrophic factors and decreasing neuronal stress. During the rapid progression (cytotoxic phase), the motor neuron becomes damaged, and the inflammatory response becomes harmful. Created with BioRender.com.

Foran extended period, neuroinflammation was considered only a consequence of motor neuron deathNowadays, it is established as an important factor for several neurodegenerative diseases. Neuroinflammation is mediated by activated glial cells and infiltrating lymphocytes, accompanied by the production of pro-inflammatory cytokines and neurotoxic or neuroprotective molecules [160]. During the initial periods of disease progression (neuroprotective phase), the immune system acts as a protector with glia and T cells, especially M2 macrophages/microglia, T helper 2 cells, and regulatory T-cells, providing anti-inflammatory factors that sustain motor neuron viability. During the period of rapid progression (cytotoxic phase), however, neuroinflammation presents a strong proinflammatory state, characterized by M1 macrophages/microglia and proinflammatory T-cells. In this phase, neuroprotective regulatory T-cells significantly decrease, and neurotoxicity predominates (Fig. **4**) [161]. Numerous studies have highlighted the relationship between disease progression and markers of neuroinflammation [162]. Post-mortem analysis of cerebrospinal fluid and spinal cord samples in ALS cases revealed increased microglial activation and lymphocyte permeation [163, 164]. Other studies demonstrate that astrocytes have toxic properties, contributing to motorneuron death, while T-lymphocytes control the microglial response in a neuroprotective mode [161, 165]. These and various other mechanisms related to neuroinflammatory processes have been extensively described [162, 166]. Additionally, several compounds with anti-inflammatory properties have been reported to enhance motor neuron survival in transgenic mice.

**Fig. (5):**
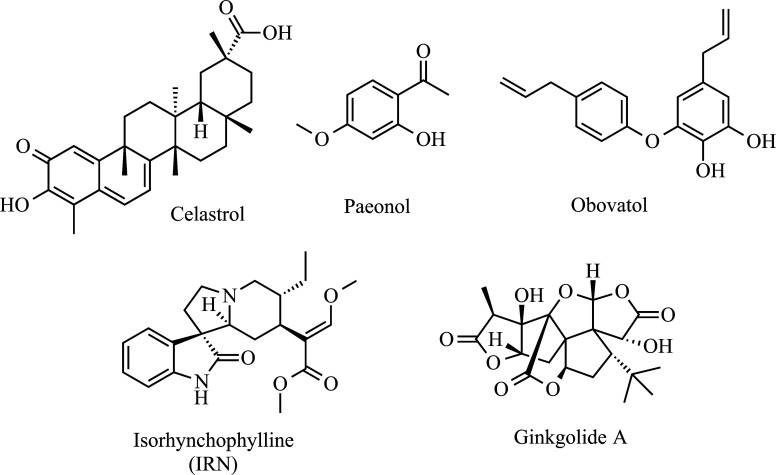
Anti Neuroinflammatory agents in ALS: Celastrol, Paeonol, Obovatol, IRN, Ginkgolide.

Some agents against OS are also frequently reported as modulators of neuroinflammatory processes, such as the aforementioned curcumin [167-171] and resveratrol [172, 173]. In addition to these, other anti-inflammatory and anti-oxidative agents will be mentioned herein (Fig. **5**). The first of these is Celastrol, which has already been reported in studies on its therapeutic potential in traditional Chinese medicine [174]. This triterpenoid pigment is extracted from *Tripterygium wilfordii* Hook F. and it was previously mentioned as an inhibitor of cancer cell proliferation, in addition to acting in the suppression of autoimmune and anti-inflammatory processes [175]. Kiaei *et al.* administered celastrol in the diet of SOD1^G93A^ mice from their 30^th^ day of life. The treatment promoted improvement in motor performance and delayed the onset of the disease. At the cellular level, lumbar spinal cord neuron cell counts demonstrated a protective effect. Additionally, celastrol down-regulated the expression of TNF-*α* and iNOS and the immunoreactivity of CD40 and GFAP (a marker of microglia and astrocytes, respectively) [176]. Later, another group reported that the excessive production of NO, TNF-*α,* and IL-1*β* induced by lipopolysaccharide in BV-2 cells was inhibited by celastrol. The underlying mechanisms associated were the inhibition of ERK1/2 MAPK phosphorylation and the DNA binding activity of NF-κB [177]. More recently, celastrol has also attenuated cadmium-induced neuronal apoptosis *via* the calcium-dependent Akt/mTOR pathway [178]. Paeonol is another important antioxidant and anti-inflammatory agent [179]. In a model of injury based on glutamate-induced apoptosis of pheochromocytoma cells, Paeonol prevents, in a dose-dependent manner, cell death and mitochondrial injury, in addition to reducing caspase-3 and p-ERK activity [180]. In another model, inflammation in microglia was induced by lipopolysaccharides, in which paeonol attenuated the overexpression of NO-synthase and cyclooxygenase 2, promoting the reduction of NO and prostaglandin E2 (PGE2). Additionally, paeonol reduced the production of ROS and upregulated HO-1. Also, in this work, the authors observed that in cortical neurons treated with 6-hydroxydopamine, paeonol attenuated the production of ROS, increased the activity of SOD1 and the expression of the antiapoptotic protein B-cell lymphoma 2 [181]. Most of these results were re-reported later [182].

Obovatol is a neolignan monomer and has been isolated from *Magnolia officinalis* fresh leaves [183]. In an important study conducted by Ock *et al.* in lipopolysaccharide-stimulated BV-2 microglial cells. After the administration of obovatol at a concentration of 10 mM, inhibition of NO production and microglial expression of pro-inflammatory cytokines were observed. In addition to the inhibition of multiple signaling pathways, such as NF-κB, STAT1 (signal transducers and activators of transcription 1), and MAPK (mitogen-activated protein kinase). In addition to these results, the authors confirmed that obovatol protects neurons from microglial toxicity and inhibits neuroinflammation in an *in vivo* model [184]. In this study, the molecular target of obovatol in microglia peroxiredoxin 2 (Prx2) was identified by affinity chromatography, and it was demonstrated that obovatol enhanced the ROS-scavenging activity of Prx2 *in vitro*, suppressing proinflammatory signaling pathways of microglia [184]. The importance of Prdx2 in neurodegenerative diseases has been recently reviewed [185].

In a work by Yuan *et al*. Isorhynchophylline (IRN), a component isolated from *Uncaria rhynchophylla* was presented as an attenuating agent of proinflammatory cytokines production such as TNF-*α* and IL-1*β* as well as NO in mouse N9 microglial cells, with potent inhibition of microglial activation. The authors report that the potential molecular mechanism for IRN-mediated attenuation was implicated in suppressions of iNOS protein level, phosphorylation of ERK and *p38* MAPKs, and degradation of IκB*α* [186]. Wogonin is an active ingredient from *Scutellaria* roots. This flavonoid has been shown to diminish lipopolysaccharide-induced TNF-*α*, IL-1*β*, and NO production in a dose-dependent manner. This last one was accompanied by suppression of iNOS induction and NF-κB activation in BV-2 microglia. Additionally, the inhibition of microglial activation by Wogonin promotes the reduction in microglial cytotoxicity in PC-12 cells. Finally, *in vivo,* experiments demonstrated that wogonin was protective against experimental brain injury [187].

Ginkgolide is a diterpene lactone extracted from *Ginkgo biloba* leaves that can present in forms A, B, C, J, and M. It has been proposed that Ginkgolide A and B can inhibit NO production in lipopolysaccharide-stimulated microglia [188]. Several studies report the role of this class in the modulation of neuroinflammation caused by neurodegenerative processes [189-192]. In addition, Chrysin and inflexin have also been reported as potential agents in neuroinflammation [193, 194].

## 4 EXCITATORY AMINO ACID TOXICITY AND ALS

**Fig. (6):**
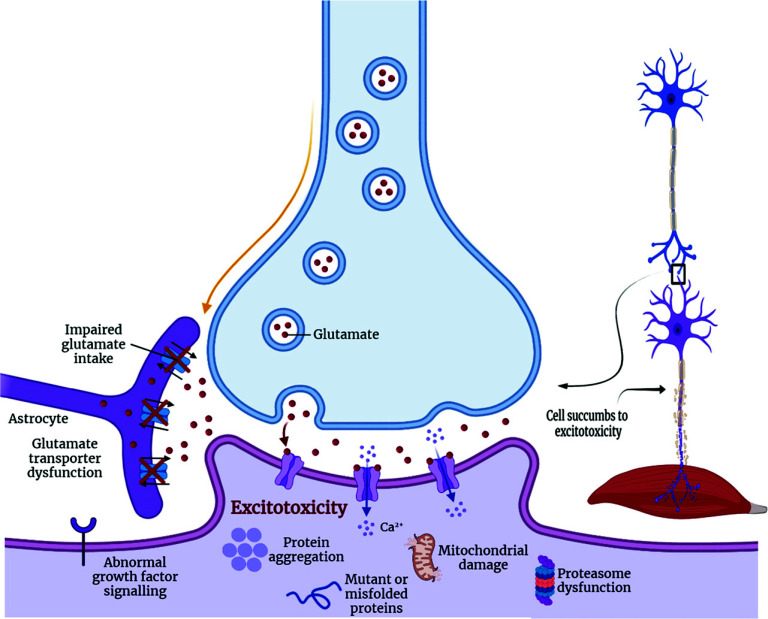
Glutamatergic transmission in ALS under hyperexcitability conditions. The release of glutamate by the presynaptic neuron stimulates the receptors on the postsynaptic neuron generating excitatory postsynaptic potentials. In ALS, presynaptic hypersensitivity causes excessive glutamate release. Created with BioRender.com.

The amino acid glutamate plays an important role within the CNS, acting as an excitatory neurotransmitter in addition to protein biosynthesis [195]. When released into the synaptic cleft, glutamate activates a family of ligand-gated ion channels called ionotropic receptors (named NMDA, AMPA, and KA). Upon termination of excitatory activity, glutamate is reuptake by specific Na^+^-dependent systems, located mainly in surrounding astrocytes, known as excitatory amino acid transporter-2 (EAAT-2) in humans and glutamate transporter-1 in rodents (GLT-1). Finally, glutamate is converted to glutamine, which has no neurotransmitter properties [196]. The excitotoxicity event is caused by excessive and unregulated activation of glutamate receptors. Prolonged exposure of these receptors to high or persistently increased concentrations of glutamate can lead to cell death [197]. Receptor hypersensitivity causes an influx of calcium through ionotropic receptors, leading to the activation of degradative enzymes, including phospholipase A2, proteases, and iNOS, which directly cause cell death and tissue damage. Elevations in intracellular calcium can disrupt mitochondrial function, leading to free radical production and impaired ATP production (Fig. **6**) [198]. The relationship between excitotoxicity and ALS can be exemplified by the finding that the cerebrospinal fluid of patients with the disease had three-fold higher concentrations of glutamate, aspartate, *N*-acetyl-aspartyl glutamate, and *N*-acetyl aspartate when compared to healthy controls [199]. In addition, increased plasma glutamate levels have been reported in ALS patients [200], as well as decreased neurotransmitter uptake and reduced levels of EAAT-2 transporter expression [201]. The pathogenic role of glutamate is even more evident as the only current treatment for ALS, riluzole, is based on glutamatergic activity in the CNS.

**Fig. (7):**
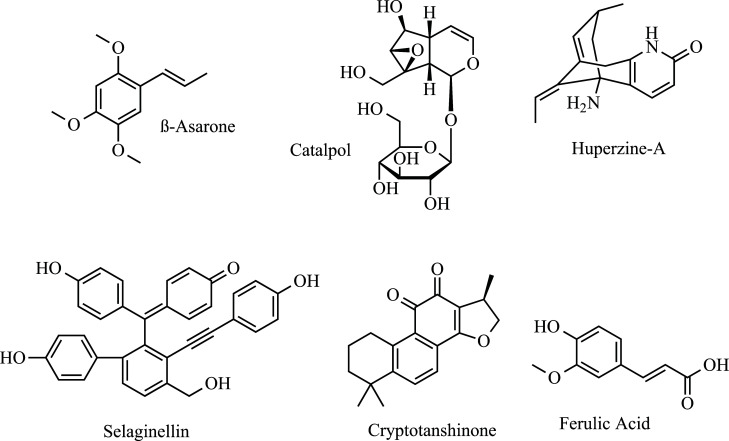
Anti excitatory amino acid agents in ALS: *β*-Asarone, Catalpol, Huperzine-A, Selaginellin, Cryptotanshinone, and Ferulic Acid.

Several natural products are reported to act on the mechanism of excitotoxicity (Fig. **7**). β-Asarone can be isolated from *Acorus tatarinowii* Schott of *Araceae* plants. A study conducted by Cho *et al.* demonstrated that *β-*asarone exhibits neuroprotective action against NMDA or glutamate-induced excitotoxicity by blocking NMDA receptor function [202]. This result was later reported again, in addition to promoting decreased LDH leakage [203]. Catalpol is an iridoid glucoside that is isolated from the roots of *Rehmannia glutinosa*. Using a cell line of apoptosis induced by H_2_O_2_, it was observed that catalpol was able to suppress the down-regulation of Bcl-2, up-regulation of *Bax,* and the release of mitochondrial cytochrome *c* to the cytosol, in addition to attenuating the activation of caspase-3, PARP cleavage, and protect against apoptosis [204]. Huperzine A (Hup-A), an alkaloid from *Huperzia serrata*, reduces glutamate-mediated Ca^2+^ signaling [205]. Hup-A has been shown to attenuate excitatory amino acid toxicity by blocking the NMDA ion channel and subsequent mobilization of Ca^2+^ at or near PCP and MK-801 ligand sites [206]. Hemendinger *et al.* proposes that Hup-A modulates Ca^2+^ levels through the Cdk5 activation pathway, which activates anti-apoptotic factors [207]. Selaginellin, a pigment extracted from *Saussurea pulvinata*, acts against glutamate-induced toxicity in PC-12 cells, reducing ROS levels and regulating the *klotho* gene [208]. Cryptotanshinone is a derivative from *Salvia miltiorrhiza* acting on glutamate-induced toxicity, protecting primary cortical neurons from neurotoxicity through activation of the phosphoinositide 3-kinase/Akt (PI3K/Akt) pathway [209, 210].

Ferulic acid is a compound found in a variety of herbs and is of neuropharmacological interest as it readily penetrates the BBB [211]. In a culture of cortical neurons with glutamate-induced apoptosis, sodium ferulate activated caspase-3 protein expression and PARP cleavage and inhibited the upregulation of glutamate-induced *μ*-calpain protein level. The mechanism appears to be *via* the PI3K/Akt/p70S6K and MEK/ERK1/2 pathways. In addition, there was inhibition of glutamate-induced reduction in Bcl-2 expression [212]. It was later reported that this cinnamic derivative reduced hippocampal neuronal apoptosis and oxidative stress and protected PC-12 cells against the influx of Ca^2+^, malondialdehyde, and production of glutathione peroxidase. The mechanism appears to be inhibition of the Toll-like receptor/myeloid differentiation factor 88 (TLR4/MyD88) signaling pathway [213]. Recently, ferulic acid has been associated with a protective effect against H_2_O_2_-induced apoptosis through the inhibition of phosphorylation of the extracellular signal-regulated kinase (ERK) [214]. Finally, some species can be cited as potential candidates for treatment due to glutamate excitotoxicity, such as saponins from *Acanthopanax senticosus* [215] and *Sea cucumber* (morina root) [216].

## 5 CALCIUM CYTOTOXICITY AND ALS

**Fig. (8):**
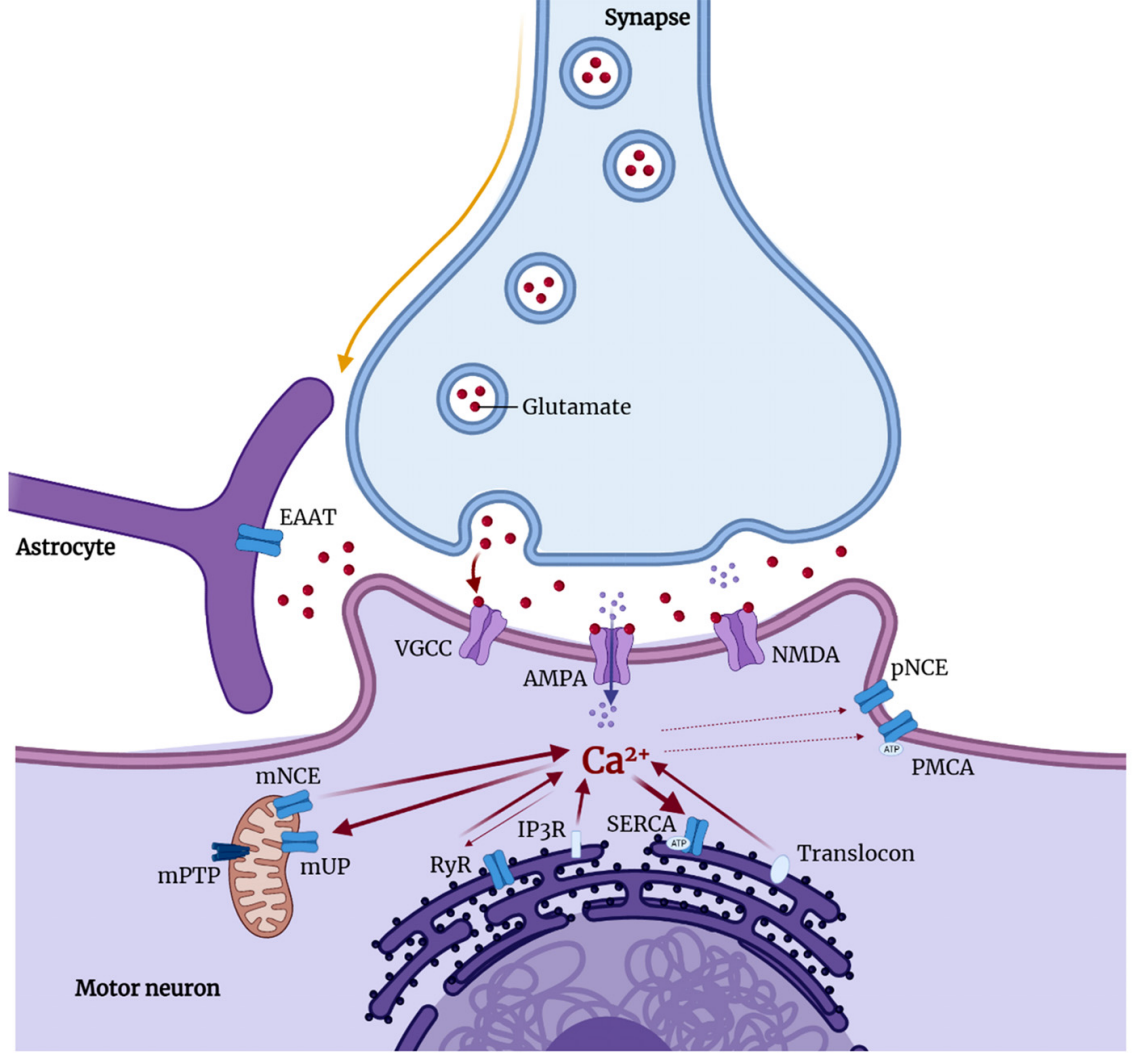
The endoplasmic reticulum mitochondria Ca^2+^ cycle and receptors involved in calcium transport.

A particular feature of ALS-affected motor neurons is related to their vulnerability to calcium overload, especially due to the high expression of Ca^2+^ permeable AMPA receptors [217, 218]. As already mentioned in excitatory amino acid toxicity, disturbances in Ca^2+^ homeostasis and protein folding are essential features of neurodegeneration since the correct protein folding is driven by folding proteins regulated by intracellular levels of Ca^2+^ [219]. Under physiological conditions, the release of Ca^2+^ in the endoplasmic reticulum is controlled by ryanodine receptors (RyR), channels controlled by inositol 1,4,5-triphosphate receptors (IP3R), and the translocon [220]. Internal control is performed by the Sarco/endoplasmic reticulum Ca^2+^ATPase (SERCA) and in the plasma membrane by sodium/Ca^2+^ and Ca^2+^ATPase. In mitochondria, Ca^2+^ uptake is performed by mUP, also controlled by cytosolic Ca^2+^ and by Ca^2+^/calmodulin. Ca^2+^ in the mitochondria can be ejected back into the cytosol *via* Na^+^/Ca^2+^ and 2H^+^/Ca^2+^ exchangers. When the concentration of Ca^2+^ within the mitochondria rises to a considerable level, the mPTP channel opens, leading to cell death by either apoptosis or necrosis [219]. Abnormalities of Ca^2+^ homeostasis, endoplasmic reticulum, and mitochondria are associated with motor neuron toxicity in ALS (Fig. **8**) [221]. It is also known that both protein misfolding and Ca^2+^ overload can induce apoptosis through Bcl-2-dependent mechanisms [221].

**Fig. (9):**
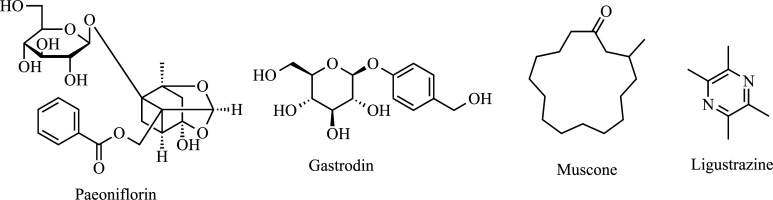
Anti-calcium cytotoxicity compounds: Paeoniflorin, Gastrodin, Muscone, and Ligustrazine.

Paeoniflorin (Fig. **9**) is the main component of *Paeoniae radix* and appears to play an important role in neuroprotection. Mao *et al.* reported that glutamate-induced neurotoxicity in PC-12 cells was evaluated. It was observed that paeoniflorin increased cell viability, inhibited apoptosis, decreased intracellular ROS and malondialdehyde levels, and increased SOD1 activity. Additionally, the treatment enabled a reduction in the Ca^2+^ overload [222]. Later, the same group demonstrated that the monoterpene glycoside decreased lactate dehydrogenase release in NMDA-treated PC-12 cells [223, 224]. Gastrodin (Fig. **9**), another plant-derived compound, has been investigated for its ability to traverse the blood-brain barrier (BBB). This *Gastrodia elata* derivative showed potential in reducing intracellular Ca^2+^ levels *via* voltage-gated Ca^2+^ channels [225]. Muscone (Fig. **9**), derived from natural musk, inhibited Glu-induced apoptosis in PC-12 cells by a mechanism related to the inhibition of intracellular Ca^2+^ overload and maintenance of mitochondrial membrane potential [226]. Finally, Ligustrazine (Fig. **9**) demonstrated inhibitory effects on *L*-type calcium current in SH-SY5Y human neuroblastoma [227].

## CONCLUSION

While there is still no effective therapy for curing ALS, using herbs and plant-based products has become a promising alternative (Table **1**). Traditional Chinese medicine has brought several complementary possibilities for treating the disease and has contributed significantly to the search for new modulators of the disease. Through what has been exposed, it is possible to understand that the treatment of ALS can benefit from herbal medicine. Additionally, tests with monomers or active substances isolated *in vivo* and *in vitro* must be performed; the mechanisms must be conclusively elucidated, and, in the future, clinical trials will be important for the safe clinical use of these substances.

## Figures and Tables

**Table 1:** Summary of natural compounds and activities related to the treatment of amyotrophic lateral sclerosis (ALS).

**Class of Pharmacological ** **Activity and Compound Name**	**Evidence of Pharmacological Activity Related to ALS**
**Oxidative Stress**	
Curcumin and Dimethoxy Curcumin (DMC)	Activate Nrf2; protect cells against stress through the removal of free radicals and detoxication; eliminate the excitability induced by TDP-43. Inhibits DTT-induced SOD1 fibrillation and favors the formation of smaller and disordered SOD1 aggregates, reduction in SOD1-mediated toxicity; assessed in *In vivo* and *in vitro* studies, with clinical trials.
Resveratrol	Up-regulating SIRT1 expression in the mutant SOD1^G93A^-bearing motor neuron-like culture model of ALS, improving the cell viability and ATP levels and preventing cell apoptosis.
Epigallocatechin gallate (EGCG)	Inhibits most types of the apoptotic cell by up-regulation of Bcl-2, downregulating the Bax gene; protecting against lipid peroxidation; preventing OS-induced death of mutant SOD1; upregulating PI3K/Akt and GSK-3 pathways in G93A mutant cells; assess in *In vivo* and *in vitro* studies.
7,8-dihydroxyflavone (7,8-DHF)	Enhanced neuromuscular transmission *via* TrkB activation in diaphragm muscle preserves spinal motor neuron count, and dendritic spines; assessed in *In vitro* studies.
Quercetin	Prevents aluminum-induced translocation of cytochrome c, upregulates Bcl-2, down-regulates Bax, p53, and caspase-3 activation, and reduces DNA fragmentation; assess in *In vivo* and *in vitro* studies.
Firestin	Increased the expression of phosphorylated ERK, increased the expression of antioxidant factors, and reduce the levels of mutant and wild-type SOD1.
Ampelopsin	Prevented H_2_O_2_-induced *p38* activation; Upregulation of HO-1 due to the activation of ERK and Akt signaling pathways; assessed in *In vitro* and *in vivo* studies.
Vitamin E	Using it as an adjuvant treatment with riluzole allowed us to verify changes in the levels of biochemical markers for oxidative stress; assessed in *In vivo* studies.
Astragaloside IV	Attenuate the H_2_O_2_-induced loss; Decrease apoptosis and attenuate ROS overproduction; Protects microglia from lipopolysaccharide-evoked death and down-regulated the release of pro-inflammatory mediators IL-1*β*, IL-6, TNF-*α,* and NO, and the expression of TLR4, MyD88, and NF-κB.
Madecassoside	Reduce neuronal damage caused by OS and inhibit apoptosis; reduce neurotoxicity, cognitive impairments, and the production of inflammatory cytokine agents (IL-1β, TNF-α, and IL-6 by activation of Nrf2 signaling); assessed in *in vitro* and *in vivo* studies.
Morronoside	Inhibitory effects on ROS formation and activation of caspase-3 and 9, up-regulate Bcl-2 in SH-SY5Y cells of a model of H_2_O_2_-induced toxicity. Reduce the intracellular accumulation of Ca^2+^ and decrease the mitochondrial membrane potential caused by the addition of H_2_O_2_.
Picroside-II	Increase in neurite outgrowth in PC-12 cells. Enable an increase in cell viability and a reduction in glutamate-induced ROS; assess in *In vitro* and *In vivo* studies.
Dially Trissufide	DATS treatment activated HO-1. Cause activation of Nrf2 and Nrf2 target gene in rat spinal cord, protected motor neurons against glutamate-induced excitotoxicity. Induces neuronal autophagy and lysosomal clearance of TDP-43 and C-terminal TDP-43 fragments.
**Neuroinflammation**	
Celastrol	Down-regulates the expression of TNF-*α* and iNOS and the immunoreactivity of CD40 and GFAP; Inhibition of excessive production of NO, TNF-*α,* and IL-1*β* induced by lipopolysaccharide in BV-2 cells; attenuates cadmium-induced neuronal apoptosis *via* the calcium-dependent Akt/mTOR pathway.
Paeonol	Prevent cell death and mitochondrial injury; Reduces caspase-3 and p-ERK activity; Attenuates the overexpression of NO-synthase and cyclooxygenase 2; Reduced the production of ROS-upregulated HO-1.
Obovatrol	Inhibition of NO production, microglial expression of pro-inflammatory cytokines, multiple signaling pathways (NF-κB, STAT1, and MAPK); Protect neurons from microglial toxicity; inhibit neuroinflammation; assess in I*n vivo* studies.
Isorhynchophylline	Attenuates proinflammatory cytokines production such as TNF-*α* and IL-1*β* as well as NO in mouse N9 microglial cells; Suppression of iNOS protein level, phosphorylation of ERK and *p38* MAPKs, and degradation of IκB*α.*
Wogonin	Diminishes lipopolysaccharide-induced TNF-α, IL-1β, and NO production in a dose-dependent manner; Promotes the reduction in microglial cytotoxicity in PC-12 cells; Protective against experimental brain injury (*in vivo*).
Ginkgolide A	Inhibits NO production in lipopolysaccharide-stimulated microglia; Modulation of neuroinflammation caused by neurodegenerative processes.
**Excitatory Amino Acid Toxicity**	
β-asarone	Neuroprotective action against NMDA or glutamate-induced excitotoxicity by blocking NMDA receptor function; Promotes a decrease in LDH leakage.
Catalpol	Suppress the down-regulation of Bcl-2 and up-regulation of *Bax;* Release mitochondrial cytochrome *c* to the cytosol; Attenuates the activation of caspase-3 and PARP cleavage and protects against apoptosis.
Huperzine-A	Reduces glutamate-mediated Ca^2+^ signaling; Attenuates excitatory amino acid toxicity; Modulates Ca^2+^ levels through the Cdk5 activation pathway.
Selaginellin	Acts against glutamate-induced toxicity in PC-12 cells, reducing ROS levels and regulating the *Klotho* gene.
Cryptotanshinone	Acts on glutamate-induced toxicity, protecting primary cortical neurons from neurotoxicity through activation of the phosphoinositide 3-kinase/Akt.
Ferulic Acid	Activated caspase-3 protein expression and PARP cleavage and inhibited the upregulation of glutamate-induced *μ*-calpain protein level; Inhibition of glutamate-induced reduction in Bcl-2 expression.
**Anti-calcium Cytotoxicity**	
Paeoniflorin	Increases cell viability, inhibits apoptosis, decreases intracellular ROS and malondialdehyde levels, and increases SOD1 activity.
Gastrodin	Reduces intracellular Ca^2+^ level *via* voltage-gated Ca^2+^ channels.
Muscone	Inhibits Glu-induced apoptosis in PC-12 cells by a mechanism related to inhibition of intracellular Ca^2+^ overload and maintenance of mitochondrial membrane potential.
Ligustrazine	Inhibits *L*-type calcium current in SH-SY5Y human neuroblastoma.

## LIST OF ABBREVIATIONS

ALS: Amyotrophic Lateral Sclerosis
BBB: Blood-brain Barrier
CNS: Central Nervous System
DATS: Diallyl Trisulfide
DMC: Dimethoxy Curcumin
EGCG: Epigallocatechin Gallate
HT: Hydroxytyrosol
NO: Nitric Oxide
OS: Oxidative Stress
RNS: Reactive Nitrogen Species
ROS: Reactive Oxygen Species
RyR: Ryanodine Receptors
